# Opportunistic Osteoporosis Screening in Breast Cancer Using AI-Derived Vertebral BMD from Routine CT: Validation Against QCT and Multivariable Diagnostic Modeling

**DOI:** 10.3390/jcm15020512

**Published:** 2026-01-08

**Authors:** Jiayi Pu, Wenqin Zhou, Miao Wei, Wen Li, Yan Xiao, Jia Xie, Fajin Lv

**Affiliations:** 1 Department of Radiology, The First Affiliated Hospital of Chongqing Medical University, Chongqing 400016, China; 2 Department of Radiology, Children’s Hospital of Chongqing Medical University, Chongqing 400014, China; 3 Department of Radiology, Yubei District Traditional Chinese Medicine Hospital, Chongqing 401120, China; 4 Department of Radiology, Bishan Hospital of Chongqing Medical University, Chongqing 420760, China; 5 Department of Radiology, Beibei District Traditional Chinese Medicine Hospital, Chongqing 400700, China

**Keywords:** breast cancer, artificial intelligence, opportunistic screening, bone mineral density (BMD), quantitative computed tomography (QCT), osteoporosis, deep learning

## Abstract

**Background/Objectives**: Breast cancer survivors face elevated risk of treatment-related bone loss, yet routine bone health assessment remains underutilized. Opportunistic bone density extraction from routine CT may address this gap. This study validated AI-derived vertebral bone mineral density (AI-vBMD) from non-contrast thoracoabdominal CT for osteoporosis screening and assessed its diagnostic value beyond clinical variables. **Methods**: This retrospective study included 332 breast cancer patients; AI-vBMD was successfully extracted in 325 (98%). Quantitative CT (QCT) served as reference standard. Agreement between AI-vBMD and QCT-vBMD was assessed using Pearson correlation, Bland–Altman analysis, and weighted kappa for QCT-defined osteoporosis (<80 mg/cm^3^). Nested logistic regression models compared a clinical model with and without AI-vBMD. Discrimination [area under the curve (AUC)], calibration, and clinical utility [decision-curve analysis (DCA)] were evaluated. **Results**: AI-vBMD showed strong correlation with QCT-vBMD (r = 0.98, *p* < 0.001), minimal bias (mean difference +1.82 mg/cm^3^), and excellent agreement for osteoporosis classification (weighted κ = 0.90). AI-vBMD alone achieved excellent discrimination for osteoporosis (AUC = 0.986). Integrating AI-vBMD into the clinical model yielded significantly higher diagnostic performance (AUC 0.988 vs. 0.879; *p* < 0.001) and demonstrated superior net benefit across relevant decision thresholds. **Conclusions**: AI-derived vertebral BMD from routine CT serves as a reliable QCT-aligned imaging biomarker for opportunistic osteoporosis assessment in breast cancer patients and adds significant incremental diagnostic value beyond clinical information alone.

## 1. Introduction

Breast cancer is the most common malignancy among women worldwide, and advances in cancer therapy have substantially improved long-term survival [1,2]. However, treatment-induced bone loss has emerged as a major survivorship issue. Aromatase inhibitors, chemotherapy-induced ovarian suppression, and concomitant corticosteroids collectively disrupt bone homeostasis and accelerate bone turnover [3,4,5], resulting in increased risk of osteoporosis and fragility fractures [6]. Despite clinical practice guidelines recommending bone mineral density assessment for women receiving aromatase inhibitor therapy [7], routine bone health screening remains suboptimal in oncology practice, underscoring the need for scalable approaches to osteoporosis risk assessment in this vulnerable population.

Current bone mineral density (BMD) assessment relies primarily on dual-energy X-ray absorptiometry (DXA), which provides areal BMD with minimal radiation exposure [8]. However, DXA measures two-dimensional projections, cannot distinguish trabecular from cortical bone, and is susceptible to artifacts from degenerative changes and vascular calcifications [9,10], potentially leading to BMD overestimation. Quantitative computed tomography (QCT) offers volumetric BMD (vBMD) measurement and selectively assesses metabolically active trabecular bone [11], but requires phantom calibration and is not routinely performed in oncology settings [12].

Breast cancer patients routinely undergo chest and abdominal CT for staging and surveillance, enabling opportunistic osteoporosis screening without additional radiation exposure. Prior studies have shown that CT attenuation-based analyses can reliably estimate BMD and fracture risk [13,14,15], but most approaches remain dependent on manual or semi-automated region of interest (ROI) delineation, limiting reproducibility and clinical scalability. Artificial intelligence (AI) offers fully automated, phantomless solutions by enabling vertebral detection, segmentation, and BMD quantification. Recent studies have demonstrated strong agreement between AI-derived vertebral BMD and QCT in general populations [16,17], yet rigorous validation in high-risk oncology cohorts remains limited. Furthermore, whether AI-derived BMD provides incremental diagnostic value beyond clinical variables has not been established.

In this study, we evaluated a commercially available deep-learning tool that automatically extracts vertebral volumetric BMD from routine non-contrast CT, with output calibrated to match QCT physical units (AI-vBMD, mg/cm^3^). Our objectives were as follows: (1) to validate diagnostic accuracy of AI-vBMD against QCT for osteoporosis identification in breast cancer patients; (2) to assess whether AI-vBMD adds diagnostic value beyond clinical and biochemical variables through multivariable modeling; and (3) to evaluate clinical utility using decision-curve analysis (DCA).

## 2. Materials and Methods

### 2.1. Study Design and Population

This retrospective, single-center study included 332 consecutive female patients with histologically confirmed primary breast cancer who underwent non-contrast thoracoabdominal CT between April 2022 and December 2023 at the First Affiliated Hospital of Chongqing Medical University. Inclusion criteria were: (i) surgical resection of the primary tumor and (ii) availability of clinical, biochemical, and pathological data required for multivariable modeling. Exclusion criteria were: (i) prior spinal surgery (e.g., vertebroplasty or metallic fixation); (ii) vertebral fracture (including compression fracture) or severe structural deformity (including severe scoliosis and significant degenerative changes) that would interfere with accurate L1–L3 segmentation or density measurement; (iii) vertebral tumor involvement; (iv) known metabolic bone disorders (e.g., primary hyperparathyroidism); and (v) imaging artifacts affecting assessment of L1–L3 vertebral levels. Clinical and biochemical variables included age, body mass index (BMI), serum uric acid, estimated glomerular filtration rate (eGFR), and bone and mineral metabolism markers (serum calcium, magnesium, phosphorus, 25-hydroxyvitamin D [25(OH)D], bone-specific alkaline phosphatase [BALP], calcitonin and parathyroid hormone [PTH]). Pathological data included breast cancer molecular subtype (Luminal A, Luminal B, HER2-positive, or triple-negative). The study was approved by the institutional review board of our hospital, and the requirement for informed consent was waived owing to its retrospective design.

### 2.2. CT Image Acquisition and QCT Measurement

All CT examinations were performed on a dual-source CT system (SOMATOM Force, Siemens Healthineers, Erlangen, Germany) using a standard non-contrast thoracoabdominal protocol that is routinely applied in our oncologic imaging practice. Scan parameters were as follows: detector collimation 192 × 0.6 mm, tube voltage 120 kVp, automatic tube current modulation, and gantry rotation time 0.25 s. Images were reconstructed with a medium soft-tissue kernel (Br40) at 1.0 mm slice thickness and 1.0 mm intervals, with a 512 × 512 matrix and a 32 cm field of view.

QCT-derived vertebral vBMD was measured using dedicated software (QCT Pro Model 4, Version 6.1, Mindways Software, Austin, TX, USA). To ensure system stability and measurement accuracy, phantom calibration was performed weekly, and air calibration was conducted daily to correct for potential scanner drift, strictly adhering to the manufacturer’s instructions and ISCD guidelines. At each vertebral level from L1 to L3, a semi-automated ROI was placed within the central trabecular compartment, avoiding cortical bone, the basivertebral venous plexus, and focal lesions. The mean vBMD across L1–L3 served as the diagnostic reference. Diagnostic categories followed the International Society for Clinical Densitometry criteria [12,18]: osteoporosis, vBMD < 80 mg/cm^3^; osteopenia, 80 ≤ vBMD < 120 mg/cm^3^; and normal BMD, vBMD ≥ 120 mg/cm^3^. Osteoporosis and osteopenia were collectively classified as low BMD (vBMD < 120 mg/cm^3^).

### 2.3. AI-Derived Vertebral BMD Measurement

AI-derived vertebral BMD assessment was performed using a commercially available, fully automated, phantomless BMD analysis software (Version 2.11.0, Huiying Medical Technology, Beijing, China). According to the manufacturer, the software was developed and validated on multi-vendor datasets to support cross-platform deployment. The software automatically performs vertebral recognition, segmentation, and quantification of mean trabecular CT attenuation. For AI-vBMD extraction, only the patient images from the non-contrast thoracoabdominal CT series were processed; the external calibration phantom was not used by the commercial software. AI-vBMD values (mg/cm^3^) are generated through an internal calibration algorithm that maps CT attenuation to volumetric BMD.

All segmentation outputs were independently reviewed by two radiologists to confirm correct vertebral labeling and segmentation accuracy, with discrepancies resolved by consensus. Patients with unresolved segmentation failure or mislabeling at vertebral levels used for AI-vBMD calculation (L1–L3) were excluded from AI-vBMD analyses. After quality control, AI-vBMD values were successfully obtained in 325 patients, with seven exclusions due to segmentation failure. For categorical analyses, AI-vBMD-based bone-status groups were defined using the same thresholds as for QCT-vBMD.

### 2.4. Statistical Analysis

Statistical analyses were performed to address three primary objectives: (1) to evaluate the correlation and agreement between AI-vBMD and QCT-vBMD; (2) to quantify the independent and incremental value of AI-vBMD when integrated with clinical and biochemical variables in multivariable models; and (3) to assess model calibration and clinical utility. The primary binary outcome for modeling was osteoporosis, defined according to the QCT-based classification described in Section 2.2. Low BMD (osteopenia plus osteoporosis) was used as a secondary combined endpoint in additional receiver operating characteristic (ROC) analyses of AI-vBMD.

All analyses were conducted using R software (version 4.3.2; R Foundation for Statistical Computing, Vienna, Austria). Continuous variables were summarized as mean ± standard deviation and categorical variables as counts and percentages. Differences across QCT-based bone-status groups were assessed using the Kruskal–Wallis test for continuous variables and the chi-square test for categorical variables. The linear association between AI-vBMD and QCT-vBMD was evaluated using Pearson correlation, while agreement was examined using Bland–Altman analysis and weighted Cohen’s kappa. To assess the consistency of AI-vBMD performance across different vertebral levels, Pearson correlation coefficients were additionally calculated for each vertebra from T1 through L5. Diagnostic performance was assessed using ROC curve analysis, from which the area under the curve (AUC), sensitivity, specificity, and Youden index were derived. Missing data in model covariates ranged from 4.2% (eGFR) to 10.5% (molecular subtype), with most variables having <6% missingness. Missing values were handled using multiple imputation by chained equations (five imputations), assuming missing at random. While standard clinical cut-offs were used for categorical grouping as described above, optimal empirical thresholds for AI-vBMD were additionally derived using Youden’s J statistic to maximize diagnostic sensitivity and specificity relative to the QCT reference standard.

To evaluate the incremental value of AI-vBMD, five nested logistic regression models were constructed. Model 1 included baseline clinical variables (age, BMI, serum uric acid, and eGFR). Model 2 included AI-vBMD as the sole predictor. Model 3 combined the variables from Model 1 with AI-vBMD. Model 4 expanded upon Model 3 by adding bone and mineral metabolism markers (serum calcium, magnesium, phosphorus, 25(OH)D, BALP, calcitonin, and PTH). Model 5 further incorporated breast cancer molecular subtype. Molecular subtype (Luminal A, Luminal B, HER2-positive, or triple-negative) was modeled as a categorical factor using dummy variables, with Luminal A as the reference category. Covariates were selected based on biological plausibility and clinical availability: BALP was included as a marker of bone formation, calcitonin for its role in osteoclast inhibition, and serum uric acid for its antioxidative association with bone mass [19]. This nested structure allowed sequential assessment of AI-vBMD’s diagnostic value when combined with baseline clinical data (Models 1–3), bone and mineral metabolism markers (Model 4), and molecular subtype (Model 5).

Model performance was then evaluated. Discrimination was quantified using the AUC with 95% confidence intervals (CIs) calculated via the DeLong method, and incremental discrimination between nested models was tested using paired bootstrap resampling (2000 iterations) for ΔAUC. Reclassification was quantified using the continuous net reclassification improvement (NRI) and integrated discrimination improvement (IDI). Calibration was assessed using the Brier score and the Hosmer–Lemeshow goodness-of-fit test. Clinical utility was evaluated using DCA. For the final multivariable model (Model 5), odds ratios (ORs) with 95% CIs were derived, and ORs for continuous predictors were scaled to clinically meaningful increments. Internal validation of model discrimination and calibration was performed using bootstrap resampling (3000 iterations). Statistical significance was defined as a two-sided *p* < 0.05.

## 3. Results

### 3.1. Patient Characteristics

A total of 332 female patients with histologically confirmed breast cancer were included. According to QCT-based classification, 150 (45.2%) had normal BMD, 126 (38.0%) had osteopenia, and 56 (16.9%) had osteoporosis. The mean age and BMI of the overall cohort were 52.7 ± 9.9 years and 23.5 ± 2.9 kg/m^2^, respectively. Patients with osteoporosis were significantly older than those with normal BMD (63.2 ± 8.1 vs. 46.6 ± 8.3 years; *p* < 0.001) and had lower eGFR (91.4 ± 19.7 vs. 102.9 ± 16.2 mL/min/1.73 m^2^; *p* < 0.001) and higher BALP (16.9 ± 9.1 vs. 13.1 ± 5.8 U/L; *p* < 0.001). Body weight and BMI also differed between groups; however, the absolute magnitude of these differences was small, and their clinical relevance should be interpreted with caution. Serum 25(OH)D concentrations did not differ significantly between bone-status groups. Molecular subtype distribution was also similar across these groups, but subtype was retained in multivariable modeling given its influence on endocrine-therapy selection and long-term bone health [20]. Demographic and clinical characteristics are summarized in Table 1.

### 3.2. Correlation, Agreement, and Diagnostic Concordance Between AI-vBMD and QCT-vBMD

**Figure 1:**
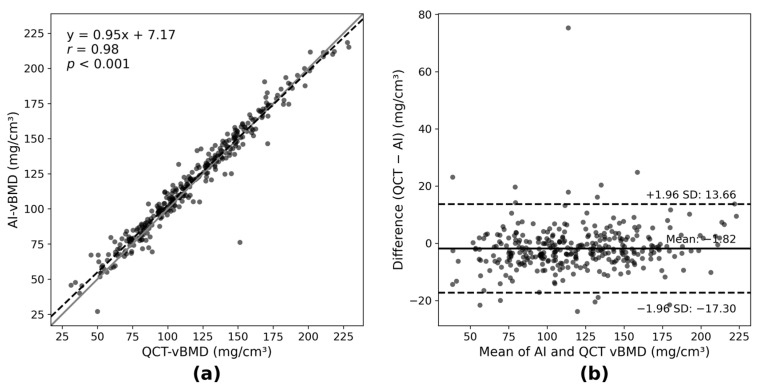
Correlation and agreement between AI-derived and QCT-derived vertebral BMD. (**a**) Scatter plot showing the correlation between AI-vBMD and QCT-vBMD across L1–L3 vertebrae. Each point represents the mean vBMD value for one patient. The solid line indicates the line of identity (*y* = *x*), and the dashed line represents the linear regression fit. (**b**) Bland–Altman plot demonstrating the agreement between AI-vBMD and QCT-vBMD. The solid horizontal line indicates the mean difference (QCT − AI), and the dashed lines indicate the upper and lower 95% limits of agreement. All BMD values are expressed in mg/cm^3^.

AI-vBMD showed strong correlation with QCT-vBMD (r = 0.98; *p* < 0.001; Figure 1a). On Bland–Altman analysis, the mean difference (QCT–AI) was −1.82 mg/cm^3^, with 95% limits of agreement from −17.30 to 13.66 mg/cm^3^ (Figure 1b). The small systematic offset, with AI-vBMD approximately 1.82 mg/cm^3^ higher than QCT-vBMD, is clinically negligible given the narrow limits of agreement. Using QCT as the reference standard for categorical classification, AI-vBMD-based osteoporosis diagnosis showed excellent agreement (weighted κ = 0.90; 95% CI, 0.87–0.94), supporting AI-vBMD as a QCT-aligned quantitative biomarker for vertebral BMD in this setting.

Level-specific correlation analysis demonstrated consistent performance of AI-vBMD across thoracic and lumbar vertebrae (T1–L5; all *p* < 0.001). Correlation coefficients progressively increased from the upper thoracic spine (T1–T3: r = 0.86–0.90) to the mid-to-lower thoracic region (T4–T11: r = 0.91–0.96), with the highest correlations observed in the T12–L3 range commonly used for diagnostic classification (r ≈ 0.99; Table A1). These findings confirm excellent concordance between AI-vBMD and QCT-vBMD across vertebral levels.

### 3.3. Diagnostic Performance of AI-vBMD

**Figure 2:**
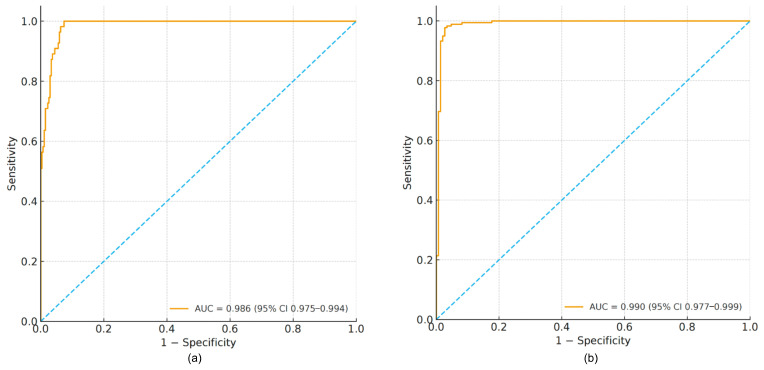
Receiver operating characteristic curves of AI-vBMD for identifying osteoporosis and low BMD. (**a**) ROC curve for osteoporosis (vBMD < 80 mg/cm^3^); AUC = 0.986 (95% CI, 0.976–0.994). The optimal threshold by Youden index was 87.04 mg/cm^3^, yielding sensitivity of 100.0% and specificity of 92.6%. (**b**) ROC curve for the combined endpoint of low BMD (osteopenia + osteoporosis; vBMD < 120 mg/cm^3^); AUC = 0.990 (95% CI, 0.977–0.999). The optimal threshold was 121.64 mg/cm^3^, yielding sensitivity of 97.8% and specificity of 97.3%. The diagonal blue dotted line indicates the line of no discrimination (AUC = 0.5), representing random classifier performance.

AI-vBMD demonstrated excellent discrimination for osteoporosis (AUC = 0.986; 95% CI, 0.976–0.994; Figure 2a). At the Youden-optimized threshold of 87.04 mg/cm^3^, sensitivity was 100.0% and specificity was 92.6% (Youden index = 0.926). For the combined endpoint of low BMD, the AUC was 0.990 (95% CI, 0.977–0.999; Figure 2b), with an optimal cut-off of 121.64 mg/cm^3^ yielding sensitivity of 97.8% and specificity of 97.3% (Youden index = 0.950).

### 3.4. Multivariable Diagnostic Modeling: Incremental Value, Calibration, and Clinical Utility

**Figure 3:**
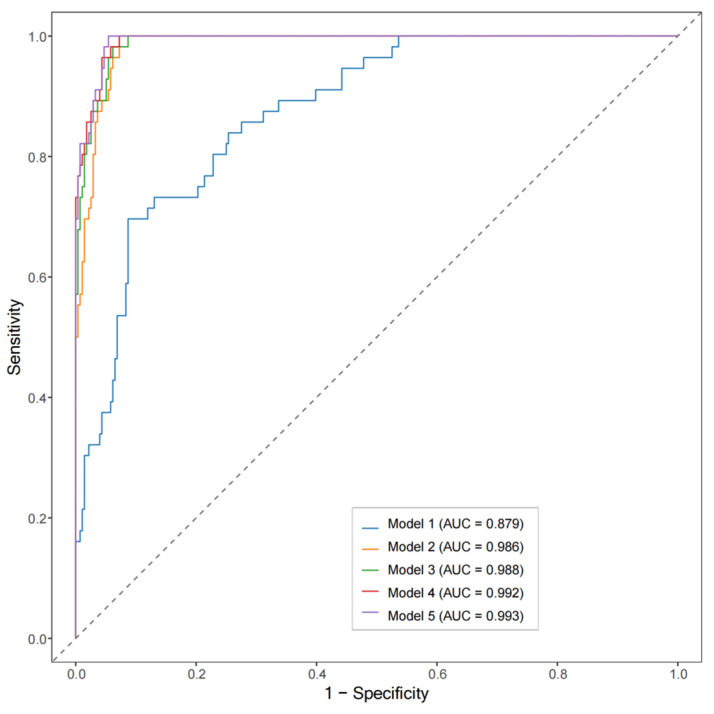
Receiver operating characteristic curves comparing nested diagnostic models for osteoporosis. ROC curves are shown for five nested logistic regression models (Models 1–5). Model compositions are detailed in Table 2. The diagonal gray dotted line indicates the line of no discrimination (AUC = 0.5), representing random classifier performance.

To optimize osteoporosis risk assessment, we evaluated five nested logistic regression models using osteoporosis as the binary outcome (Table 2; Figure 3). The AI-vBMD-only model (Model 2) achieved an AUC of 0.986. When AI-vBMD was combined with baseline clinical variables (Model 3; clinical–radiologic model), the AUC increased to 0.988, with a Brier score of 0.033. Model 3 significantly outperformed the clinical-only model (Model 1) in discrimination (ΔAUC = +0.113, *p* < 0.001) and reclassification (continuous NRI = 0.29; IDI = 0.06; both *p* < 0.01). Pairwise comparisons confirmed that the introduction of AI-vBMD provided the largest gain in discrimination, whereas subsequent expansion with biochemical markers and molecular subtype (Models 4 and 5) yielded only marginal, non-significant improvements (Table 3). Although Model 5 achieved the highest AUC (0.990), Model 3 demonstrated the best parsimony with the lowest AIC (68.5 vs. 70.2 for Model 5). This suggests that the combination of baseline clinical variables and AI-vBMD captures the majority of discriminative information, representing the most efficient model for clinical implementation.

**Figure 4:**
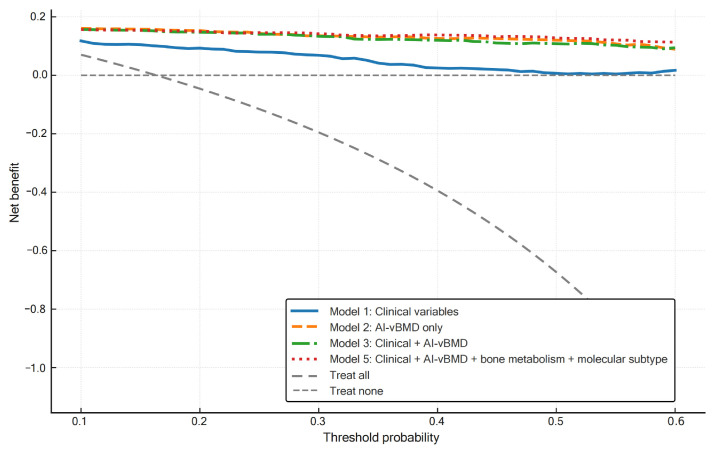
Decision-curve analysis of diagnostic models for osteoporosis. Net benefit is plotted against threshold probability for four models: Model 1 (clinical variables), Model 2 (AI-vBMD only), Model 3 (Model 1 + AI-vBMD; clinical–radiologic model), and Model 5 (Model 3 + bone and mineral metabolism markers + molecular subtype). Gray dashed curves represent the treat-all and treat-none strategies.

**Figure 5:**
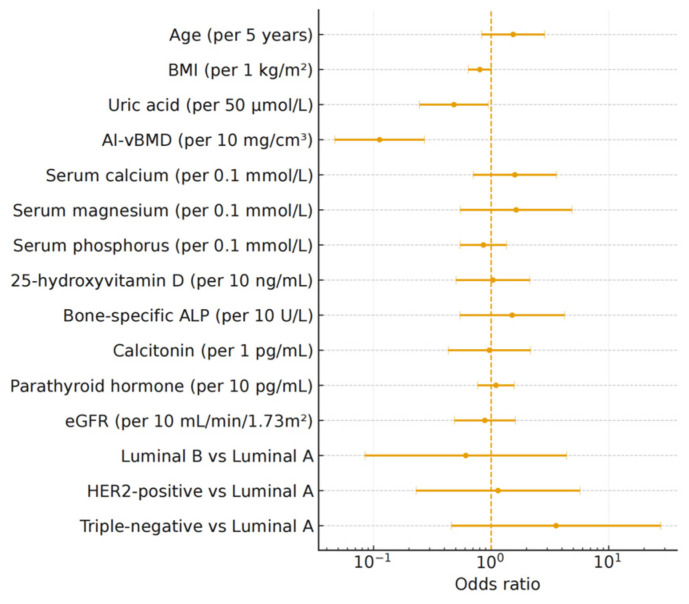
Forest plot of adjusted odds ratios in the final multivariable model (Model 5) for osteoporosis. The plot displays adjusted odds ratios (ORs) and 95% confidence intervals (CIs) for all clinical, biochemical, and molecular predictors. Continuous variables are expressed per clinically meaningful increment as indicated on the *y*-axis (e.g., AI-vBMD per 10 mg/cm^3^, age per 5 years). Molecular subtypes are shown as contrasts versus Luminal A. The vertical dashed line denotes the null value (OR = 1).

Internal validation with bootstrap resampling confirmed stable AUC estimates and statistically significant improvements compared to Model 1. Decision-curve analysis demonstrated higher net clinical benefit for all models incorporating AI-vBMD across a wide range of threshold probabilities (Figure 4). All multivariable models demonstrated good calibration. The Hosmer-Lemeshow test yielded non-significant *p*-values for all models (all *p* > 0.05), indicating acceptable alignment between predicted probabilities and observed outcomes. Brier scores ranged from 0.026 to 0.097 across models, with progressive improvement as predictors were added: Model 1, 0.097; Model 2, 0.037; Model 3, 0.033; Model 4, 0.028; and Model 5, 0.026. In the final multivariable model (Model 5), after scaling predictors to clinically meaningful increments, AI-vBMD was identified as a significant independent predictor, with each 10 mg/cm^3^ increase associated with an 88.7% reduction in the odds of osteoporosis (OR = 0.113; 95% CI, 0.047–0.270; *p* < 0.001). Odds ratios for all predictors are visualized in Figure 5.

## 4. Discussion

In this study, we validated a deep-learning–based method to extract a quantitative imaging biomarker, AI-derived vertebral BMD (AI-vBMD), from routine non-contrast CT examinations performed for breast cancer staging and follow-up in a high-risk cohort of women with breast cancer [3,4,5,6]. AI-vBMD showed excellent agreement with QCT, achieved excellent diagnostic accuracy for osteoporosis (AUC = 0.986), and significantly improved multivariable diagnostic models over clinical variables alone. In the fully adjusted model, AI-vBMD remained a significant independent predictor of osteoporosis. Taken together, the results support the use of this commercially available AI tool as a reliable, QCT-aligned measure of vertebral BMD for opportunistic osteoporosis screening in breast cancer patients, pending external and prospective validation.

Opportunistic osteoporosis screening using routine CT has long relied on vertebral attenuation in Hounsfield units (HU) as a surrogate for bone density. Although numerous studies have demonstrated its diagnostic utility, clinical implementation remains limited owing to well-recognized challenges [21,22]. The most critical limitation is the lack of standardization: heterogeneity in scanners, acquisition parameters, and reconstruction kernels leads to variability in proposed HU thresholds across cohorts. For example, Pickhardt et al. reported that for DXA-defined osteoporosis, an L1 attenuation threshold of ≤160 HU achieved 90% sensitivity, whereas ≤110 HU provided >90% specificity [13]. In an Australian cohort, Abbouchie et al. found that L1 >180 HU effectively ruled out osteoporosis or moderate osteopenia with high certainty, while >190 HU in females further decreased that probability [23], underscoring the variability of HU cut-offs across different settings and populations. Manual ROI placement introduces additional observer-dependent variability. Collectively, these limitations impede reliable clinical translation, restrict longitudinal monitoring using HU-based opportunistic screening, and limit its multicenter generalizability.

To address these challenges, deep-learning approaches have emerged for automated BMD estimation from CT, aiming to replace manual ROI placement and overcome the intrinsic limitations of HU-based surrogates. Such automation, encompassing vertebral localization, segmentation, and trabecular ROI extraction, reduces observer dependence, improves reproducibility, and supports large-scale application within clinical imaging workflows [24,25]. Several studies have shown that fully automated deep-learning pipelines enable direct estimation of vertebral BMD from conventional or low-dose CT, maintaining high consistency with QCT measurements [26,27].

Most existing work on AI-vBMD has focused on technical validation and concordance with QCT in general populations [17]. In contrast, evidence in well-defined high-risk clinical groups, such as breast cancer patients receiving systemic therapy, remains limited, and the incremental diagnostic value of AI-vBMD within multivariable clinical models has rarely been quantified. In our study, AI-vBMD provided the largest gain in discrimination when added to a clinical model, with good calibration and clear net benefit on DCA in breast cancer patients undergoing routine CT for staging or follow-up. Together, these results move beyond technical feasibility towards rigorous clinical validation, framing AI-vBMD as a unit-bearing, QCT-aligned quantitative imaging biomarker.

The strong performance of AI-vBMD primarily reflects its construct alignment with QCT and the technical robustness of deep-learning–based quantification. Unlike HU-based attenuation measures, AI-vBMD directly quantifies vertebral trabecular density on the same physical scale as QCT (mg/cm^3^), allowing results to be interpreted against established QCT thresholds. This construct alignment is reflected in the excellent correlation and minimal systematic bias observed on Bland–Altman analysis. Methodologically, the deep-learning algorithm performs automated vertebral segmentation and aggregates three-dimensional contextual information. Compared with traditional manually placed central cylindrical ROIs, this approach samples a broader trabecular region while more effectively excluding cortical bone. This automated sampling reduces susceptibility to local heterogeneity, partial-volume effects, and focal variations near endplates, thereby enhancing technical robustness. The consistent performance of AI-vBMD across vertebral levels (T1–L5), with progressively higher correlations toward the lower thoracic and lumbar spine, further supports the reliability of the automated pipeline. However, it is essential to acknowledge that robustness also depends on algorithmic stability across diverse hardware and acquisition settings. Our current validation was limited to a single scanner platform (SOMATOM Force, Siemens) with standardized non-contrast protocols. Future studies incorporating varied reconstruction kernels, slice thicknesses, contrast-enhanced protocols, and multi-vendor platforms are necessary to confirm generalizability. Notably, our derived optimal threshold for osteoporosis (87.04 mg/cm^3^) was higher than the standard QCT diagnostic cutoff (80 mg/cm^3^). This upward shift reflects two factors: first, the systematic positive bias in AI-vBMD measurements (+1.82 mg/cm^3^ relative to QCT); and second, the measurement variability evidenced by wide 95% limits of agreement. The empirically optimized threshold, derived via Youden index maximization, effectively compensates for this uncertainty, achieving perfect sensitivity in this cohort while maintaining excellent diagnostic concordance with QCT classification. Finally, this strong construct alignment reflects the inherent nature of cross-sectional osteoporosis classification, where AI-vBMD directly captures the primary physical substrate of the diagnostic standard. Consequently, AI-vBMD encompasses the majority of diagnostic variance, which likely accounts for the limited incremental discrimination provided by biochemical variables Whether these factors provide complementary prognostic value for future bone loss or incident fracture risk warrants further investigation in prospective longitudinal studies.

These findings have important clinical implications. AI-vBMD enables opportunistic bone health assessment from existing CT data without additional radiation or manual effort, offering a scalable solution for oncology populations. In breast cancer, systemic treatments such as chemotherapy and endocrine therapy can adversely affect bone metabolism, increasing the risk of osteoporosis and fragility fractures [28,29]. Younger patients often face a higher risk of premature osteoporosis compared with age-matched controls [30], whereas older patients experience compounded effects of age-related bone loss and therapy-related factors [31,32]. Notably, the magnitude of therapy-related bone loss varies by specific regimen; for instance, aromatase inhibitors are generally associated with more profound bone loss compared with tamoxifen. While granular data on individual endocrine regimens were not available for this retrospective cohort, we utilized molecular subtype as a clinical surrogate, as it fundamentally dictates the choice of systemic therapy. Although the incremental diagnostic gain of adding molecular subtype was minimal, likely because AI-vBMD already captures the resulting physical bone loss, including it in our multivariable framework indicates that AI-vBMD remains a statistically significant independent predictor of bone status after adjusting for these underlying clinical determinants. Given that vertebral fractures occur in approximately 11% of breast cancer survivors [33], early identification of low bone mass is critical. Prophylactic therapies such as denosumab or bisphosphonates have demonstrated efficacy in reducing fracture incidence [34,35,36]. However, real-world adoption of DXA screening and bone-protective management remains suboptimal, with only around half of eligible patients receiving guideline-recommended baseline DXA screening [37,38]. By integrating AI-vBMD into routine oncology imaging workflows, clinicians can systematically identify at-risk patients and implement guideline-recommended bone-protective management.

To maximize clinical utility, these findings support a pragmatic two-step implementation strategy. First, the AI-vBMD-only model (Model 2) can function as a high-throughput, fully automated triage tool integrated into radiology PACS or reporting workflows, flagging at-risk patients (e.g., those below an actionable threshold of <87 mg/cm^3^) without additional radiation or manual effort. Second, for patients identified by this initial screen or those requiring more comprehensive risk assessment, the combined clinical–radiologic model (Model 3) provides more nuanced, calibrated risk estimates. Clinicians could then act on these results by referring patients for confirmatory DXA, evaluating vertebral fracture status, or considering early prophylactic antiresorptive therapy, particularly for those receiving aromatase inhibitors. This tiered approach may facilitate systematic identification of at-risk patients who might otherwise remain undetected and support more efficient allocation of limited DXA and specialist resources.

Ethnic variability in bone density and fracture risk warrants particular consideration when interpreting opportunistic osteoporosis screening strategies. Recent work by Wáng [39] demonstrated that the conventional DXA T-score threshold of −2.5 may substantially overestimate the prevalence of osteoporosis in older Chinese women, underscoring that diagnostic cut-offs derived from one population may not be universally applicable. Similarly, Himič et al. [40] emphasized that genetic and epigenetic factors play a pivotal role in determining spinal fragility fracture risk, which are not fully captured by current standardized diagnostic criteria. In this context, our study utilized QCT-derived vBMD expressed in absolute units (mg/cm^3^) to provide a direct, three-dimensional assessment of vertebral trabecular bone. However, the optimal diagnostic thresholds identified in our Chinese cohort may still require recalibration when applied to other ethnic populations. Consequently, before broad clinical implementation, external validation in independent cohorts from diverse geographic and ethnic backgrounds will be essential to confirm the generalizability and clinical utility of AI-vBMD.

This study has several limitations. First, the retrospective, single-center design based on a Chinese breast cancer cohort limits generalizability. Diagnostic thresholds for osteoporosis and fracture risk may vary across ethnic populations, and therefore the identified vBMD cut-offs may not be directly applicable to other groups. Furthermore, as the validation was limited to a single CT scanner and standardized non-contrast protocol, the potential influence of broader imaging protocol variability was not explicitly tested. Second, detailed granular data on individual endocrine therapy regimens and duration were not available, although molecular subtype served as a clinical proxy for treatment-related risk. Third, the requirement for available clinical and biochemical data may introduce spectrum bias, and potential selection bias from AI segmentation failures (2.1%) cannot be entirely ruled out. Fourth, only one commercial AI solution was evaluated; thus, results may not be directly transferable to other AI implementations. Fifth, the primary endpoint was QCT-defined osteoporosis rather than incident fragility fractures; prospective longitudinal studies are required to establish the prognostic value of AI-vBMD for actual fracture events. Finally, because AI-vBMD is closely aligned with the physical scale of QCT, discrimination for cross-sectional classification may approach a performance ceiling, which likely accounts for the limited incremental gain observed when adding biochemical clinical variables in this validation framework.

## 5. Conclusions

AI-derived vertebral BMD (AI-vBMD) automatically extracted from routine non-contrast CT shows excellent agreement with QCT-vBMD and excellent diagnostic performance for osteoporosis in breast cancer patients. As a quantitative, QCT-aligned imaging biomarker, AI-vBMD provides a significant independent contribution in multivariable diagnostic models, and its integration with simple clinical variables modestly improves overall diagnostic performance and clinical utility. These findings support a dual-implementation strategy: automated opportunistic screening using AI-vBMD alone, and individualized risk assessment using a combined clinical–radiologic model. If confirmed through multicenter validation and prospective fracture risk prediction studies, this approach could enable scalable, low-burden bone health assessment in oncology populations without additional radiation exposure or manual effort.

## Figures and Tables

**Table 1:** Demographic and clinical characteristics of the study cohort.

Variable	Overall	QCT-Based Classification			*p* Value	AI-Based Classification		
		Normal	Osteopenia	Osteoporosis	(QCT)	Normal	Osteopenia	Osteoporosis
*N*	332	150 (45.2%)	126 (38.0%)	56 (16.9%)	-	155 (46.7%)	113 (34.0%)	57 (17.2%)
Age (years)	52.71 (9.89)	46.59 (8.26)	55.35 (6.90)	63.18 (8.10)	<0.001 ***	46.70 (8.22)	55.67 (6.72)	62.86 (7.88)
Height (cm)	157.01 (4.87)	157.42 (5.14)	157.05 (4.32)	155.83 (5.17)	0.050 *	157.40 (5.11)	157.31 (4.45)	155.44 (4.89)
Weight (kg)	58.00 (8.18)	57.32 (8.77)	59.35 (7.40)	56.78 (7.92)	0.017 *	57.10 (8.79)	59.57 (7.12)	57.53 (8.38)
BMI (kg/m^2^)	23.50 (2.93)	23.09 (3.01)	24.07 (2.82)	23.35 (2.80)	0.005 **	23.01 (3.03)	24.09 (2.73)	23.77 (2.97)
Disease duration (years)	3.74 (3.25)	3.22 (2.55)	4.36 (3.67)	3.79 (3.78)	0.035 *	3.19 (2.62)	4.41 (2.82)	3.98 (5.00)
QCT-vBMD (mg/cm^3^)	117.79 (39.32)	153.48 (24.41)	99.49 (11.30)	63.37 (11.97)	<0.001 ***	151.02 (25.50)	97.83 (11.90)	66.52 (17.45)
AI-vBMD (mg/cm^3^)	119.53 (38.24)	153.68 (24.65)	101.66 (13.08)	68.20 (12.65)	<0.001 ***	152.52 (24.13)	100.57 (10.66)	67.41 (11.48)
Uric acid (µmol/L)	299.47 (68.03)	290.56 (69.47)	313.02 (62.03)	292.39 (73.60)	0.036 *	290.35 (69.65)	310.69 (63.12)	299.90 (72.08)
eGFR (mL/min/1.73 m^2^)	97.74 (18.08)	102.87 (16.21)	94.21 (17.97)	91.37 (19.65)	<0.001 ***	102.92 (16.20)	93.89 (18.14)	91.68 (19.66)
25-hydroxyvitamin D (ng/mL)	25.62 (10.39)	24.80 (10.69)	26.31 (9.60)	26.24 (11.28)	0.437	24.52 (10.52)	27.56 (9.93)	24.64 (10.51)
Bone-specific ALP (U/L)	14.77 (7.33)	13.13 (5.83)	15.74 (7.66)	16.91 (9.13)	<0.001 ***	13.43 (6.62)	15.30 (7.04)	17.19 (9.19)
Molecular subtype, *n* (%)								
Luminal A	103 (31.0%)	42 (12.7%)	42 (12.7%)	19 (5.7%)	-	43 (13.0%)	38 (11.4%)	22 (6.6%)
Luminal B	52 (15.7%)	23 (6.9%)	22 (6.6%)	7 (2.1%)	-	24 (7.2%)	21 (6.3%)	7 (2.1%)
HER2+	93 (28.0%)	47 (14.2%)	33 (9.9%)	13 (3.9%)	-	50 (15.1%)	27 (8.1%)	15 (4.5%)
Triple-negative	48 (14.5%)	27 (8.1%)	11 (3.3%)	10 (3.0%)	-	29 (8.7%)	11 (3.3%)	8 (2.4%)
χ^2^ test for subtype across QCT groups					0.327			

Note: *p* values refer to comparisons across QCT-based bone-status groups. * *p* < 0.05; ** *p* < 0.01; *** *p* < 0.001. For some variables (e.g., BMI), statistically significant differences were accompanied by small absolute differences.

**Table 2:** Performance metrics of nested diagnostic models for osteoporosis.

Model	Predictors	*n*	AUC	AUC 95% CI	ΔAUC	ΔAUC 95% CI	ΔAUC *P*	Brier	AIC
Model 1	age, BMI, serum uric acid, eGFR	294	0.879	0.829–0.923	-	-	-	0.097	188.1
Model 2	AI-vBMD only	325	0.986	0.975–0.994	+0.110	0.067–0.161	**<0.001**	0.037	76.2
Model 3	Model 1 + AI-vBMD (clinical–radiologic model)	287	0.988	0.978–0.996	+0.113	0.070–0.161	**<0.001**	0.033	68.5
Model 4	Model 3 + bone and mineral metabolism markers *	283	0.992	0.983–0.998	+0.118	0.074–0.166	**<0.001**	0.028	73.6
Model 5	Model 4 + molecular subtype ^†^	283	0.993	0.986–0.998	+0.119	0.075–0.168	**<0.001**	0.026	77.6

* Serum calcium, magnesium, phosphorus, 25(OH)D, BALP, calcitonin, PTH. ^†^ Luminal B, HER2-positive, and triple-negative vs. Luminal A (reference category). AUC, area under the receiver operating characteristic curve; CI, confidence interval; ΔAUC, difference in AUC relative to Model 1. Bold text indicates statistical significance (*p* < 0.001). Calibration was assessed using Hosmer-Lemeshow test (all *p* > 0.05) and Brier score.

**Table 3:** Pairwise comparisons of AUC between nested models for osteoporosis.

Model Comparison	*n*	ΔAUC	ΔAUC 95% CI	*p*
Model 2 − Model 1	287	+0.110	0.066–0.158	**<0.001**
Model 3 − Model 2	287	+0.003	−0.001–0.008	0.1960
Model 4 − Model 3	283	+0.004	−0.001–0.010	0.1113
Model 5 − Model 4	283	+0.001	−0.001–0.004	0.4353

ΔAUC, difference in AUC between the two models being compared; CI, confidence interval. Bold text indicates statistical significance (*p* < 0.001). Model compositions are shown in Table 2.

## Data Availability

The data underlying this study are not publicly available due to institutional and ethical restrictions related to patient privacy, and cannot be shared outside the study institution.

## Abbreviations

The following abbreviations are used in this manuscript:

BMD	Bone mineral density
DXA	Dual-energy X-Ray absorptiometry
QCT	Quantitative computed tomography
vBMD	Volumetric BMD
ROI	Region of interest
AI	Artificial intelligence
DCA	Decision-curve analysis
eGFR	Estimated glomerular filtration rate
25(OH)D	25-hydroxyvitamin D
BALP	Bone-specific alkaline phosphatase
PTH	Parathyroid hormone
ROC	Receiver operating characteristic
AUC	Area under the curve
BMI	Body mass index
CIs	Confidence intervals
NRI	Net reclassification improvement
IDI	Integrated discrimination improvement
ORs	Odds ratios
HU	Hounsfield unit

## Appendix A

**Table A1:** Level-specific correlation and linear regression analysis between AI-vBMD and QCT-vBMD.

Vertebral Level	Pearson’s r	*p* Value	Slope (95% CI)	Intercept (95% CI), mg/cm^3^	R^2^
T1	0.864	<0.001	0.857 (0.803, 0.912)	57.814 (50.784, 64.845)	0.746
T2	0.87	<0.001	0.895 (0.841, 0.949)	38.933 (31.942, 45.924)	0.757
T3	0.896	<0.001	0.902 (0.854, 0.950)	35.398 (29.228, 41.567)	0.803
T4	0.908	<0.001	0.904 (0.860, 0.948)	33.127 (27.383, 38.871)	0.825
T5	0.927	<0.001	0.913 (0.874, 0.952)	31.366 (26.263, 36.468)	0.859
T6	0.937	<0.001	0.911 (0.875, 0.947)	29.903 (25.227, 34.579)	0.878
T7	0.948	<0.001	0.928 (0.895, 0.962)	25.366 (21.063, 29.670)	0.899
T8	0.933	<0.001	0.908 (0.871, 0.945)	26.104 (21.283, 30.925)	0.871
T9	0.947	<0.001	0.934 (0.900, 0.968)	25.037 (20.649, 29.425)	0.896
T10	0.96	<0.001	0.937 (0.908, 0.966)	25.227 (21.469, 28.984)	0.922
T11	0.946	<0.001	0.939 (0.905, 0.974)	21.342 (16.898, 25.786)	0.895
T12	0.991	<0.001	0.978 (0.964, 0.993)	7.242 (5.377, 9.107)	0.981
L1	0.995	<0.001	0.993 (0.982, 1.003)	1.579 (0.174, 2.984)	0.99
L2	0.994	<0.001	1.023 (1.010, 1.035)	−7.999 (−9.590, −6.409)	0.987
L3	0.986	<0.001	1.009 (0.991, 1.027)	−12.979 (−15.311, −10.648)	0.973
L4	0.974	<0.001	1.000 (0.975, 1.025)	−8.963 (−12.173, −5.753)	0.949
L5	0.963	<0.001	1.029 (0.997, 1.060)	−3.999 (−8.070, 0.073)	0.928

Level-specific Pearson’s correlation coefficients, linear regression parameters, and coefficients of determination (R^2^) between AI-derived vertebral bone mineral density (AI-vBMD) and quantitative CT-derived bone mineral density (QCT-vBMD). All *p* values were <0.001. Slopes close to 1.0 and intercepts near 0 indicate proportional agreement. The L1–L3 region, used for diagnostic classification in this study, demonstrated the highest correlations (r ≥ 0.99).
